# SMC1A facilitates gastric cancer cell proliferation, migration, and invasion via promoting SNAIL activated EMT

**DOI:** 10.1186/s12876-023-02850-z

**Published:** 2023-08-04

**Authors:** Yaling Liu, Xianrui Fang, Qianqian Wang, Da Xiao, Ting Zhou, Kuo Kang, Zhenyu Peng, Feng Ren, Jingyu Zhou

**Affiliations:** 1 Department of Geriatrics Surgery, The Second Xiangya Hospital, Central South University, No. 139 Renmin Road, Furong District, Changsha City, 410011 Hunan Province China; 2 Department of General Surgery, Yantai Qishan Hospital, Yantai, 264000 Shandong China; 3 Department of Oncology, The Affiliated ZhuZhou Hospital of XiangYa Medical College, Central South University, ZhuZhou, 412007 Hunan China; 4 Department of General Surgery, Shekou People’s Hospital, Shenzhen, 518000 Guangdong China

**Keywords:** Gastric cancer, Structural maintenance of chromosomes protein 1A, SNAIL, Epithelial-mesenchymal transition

## Abstract

**Background:**

Structural maintenance of chromosomes protein 1 A (SMC1A) is a crucial subunit of the cohesion protein complex and plays a vital role in cell cycle regulation, genomic stability maintenance, chromosome dynamics. Recent studies demonstrated that SMC1A participates in tumorigenesis. This reseach aims to explore the role and the underlying mechanisms of SMC1A in gastric cancer (GC).

**Materials and methods:**

RT-qPCR and western blot were used to examine the expression levels of SMC1A in GC tissues and cell lines. The role of SMC1A on GC cell proliferation, migration, invasion and epithelial-mesenchymal transition (EMT) were analyzed. Furthermore,the mechanism of SMC1A action was investigated.

**Results:**

SMC1A was highly expressed in GC tissues and cell lines. The high expression of SMC1A indicated the poor overall survival of GC patients from Kaplan-Meier Plotter. Enhancing the expression of SMC1A in AGS cells remarkably promoted cell proliferation in vitro and in vivo, migration and invasion, Conversely, knockdown of SMC1A in HGC27 cells inhibited cell proliferation, migration and invasion. Moreover, it’s observed that SMC1A promoted EMT and malignant cell behaviors via regulating SNAIL.

**Conclusion:**

Our study revealed that SMC1A promotes EMT process by upregulating SNAIL, which contributes to gastric cancer cell proliferation, migration and invasion. Therefore, targeting SMC1A may be a potential strategy to improve GC therapy.

**Supplementary Information:**

The online version contains supplementary material available at 10.1186/s12876-023-02850-z.

## Background

One of the most common digestive tract cancers, gastric cancer (GC), poses a major danger to human health [1]. Despite a recent drop in incidence, the fatality rate is still quite high. The onset and progression of GC are caused by the interplay of internal genetic variability and different external risk factors [2]. Numerous patients were in the middle or late stages when they were identified with stomach cancer since early screening had a poor detection rate, the disease was prone to invasion and metastasis, and the 5-year survival rate was exceedingly low [3]. GC is caused by a complex process involving several genes [1]. Therefore, there is an urgent need to investigate the fundamental processes of GC start-up and development in more detail.

One important component of the cohesion protein complex, which is critical for sister chromatid cohesion in chromosome dynamics, is structural maintenance of chromosomes protein 1A (SMC1A) [4]. SMC1A is essential for maintaining genomic integrity, controlling cell cycle progression, and regulating chromosome dynamics [5–7]. Recent investigations have shown that SMC1A has a role in tumorigenesis [8]. SMC1A is abundantly expressed in prostatic carcinoma, and knockdown of SMC1A might inhibit cell proliferation, growth, migration, and cancer stem-like cell features, while also improving the effectiveness of radiation treatment [9, 10]. According to Zhang et al. [11], phosphorylated SMC1A promoted the proliferation and migration of hepatocellur carcinoma cells, and its overexpression was significantly associated with worse prognostic outcomes. In colorectal cancers, SMC1A was present as extra-copies, mutations, and overexpression, and it contributes to cancer development and metastasis [12, 13]. Additionally, SMC1A overexpression was identified as an independent poor prognostic predictor in advanced colorectal cancers [14]. However, it has been reported that patients with acute myeloid leukemia who express of SMC1A poorly have a poor prognosis for survival [15]. SMC1A was shown to be correlated with patients’ survival in cases of GC [16]. SMC1A’s function and underlying mechanisms in GC remained unclear nonetheless.

In this study, we investigated the relationship between SMC1A expression levels and the predictive survival of GC patients by examining the expression of SMC1A in GC tissues and cell lines. Additionally, we assessed SMC1A’s influence and probable mechanism on biology behaviors of GC cells. Our research revealed that SMC1A promotes GC cells proliferation, migration, and invasion via activating EMT of snail family transcriptional repressor 1 (SNAI1 or SNAIL), suggesting a potential therapeutic target for GC.

## Methods

### Clinical specimens and survival analysis

A total of 20 primary stomach adenocarcinoma cancer tissues (*n* = 20) and their matched adjacent normal tissues (*n* = 20) were collected from patients who underwent surgical resection in the Department of Geriatric Surgery, the Second Xiangya Hospital, Central South University from 2018 to 2019. All specimen were diagnosed by two professional pathologists, and no patient received chemotherapy or radiotherapy prior to surgery. This study was approved by the Ethics Committee of the Second Xiangya Hospital of Central South University, and all patients have signed informed consent. The fresh tissues were fast frozen in liquid nitrogen and stored at -80℃ until use.

To investigate the correlation between SMC1A expression and the overall survival of GC patients, we employed the public data from Kaplan-Meier Plotter (http://kmplot.com/analysis/), which is capable to assess the correlation between the expression of all genes (mRNA, miRNA, protein) and survival in over 30,000 samples from 21 tumor types, including breast, ovarian, lung, & gastric cancer. Sources for the databases include GEO, EGA, and TCGA. Gene expression data and overall survival information are downloaded from GEO, EGA and TCGA. The database is handled by a PostgreSQL server, which integrates gene expression and clinical data simultaneously. To analyze the prognostic value of SMC1A, the patient samples are split into two groups basing on the median value of SMC1A expression in GC tissues. The two patient cohorts are compared by a Kaplan-Meier survival plot.

### Cell culture and transfection

Human gastric cancer cell lines AGS, HGC27, NCI-N87 and human gastric mucosal epithelial cell line GSE-1 were acquired from the Cell Bank of Chinese Academy of Sciences (Shanghai, China). AGS cells were cultured in F12K medium, while HGC27, NCI-N87 and GES-1 cells were maintained in RPMI 1640 medium. Both media were supplemented with 10% fetal bovine serum(FBS) (Gibco, USA). All cells were cultured at 37 ℃ in a humidified incubator with 5% CO_2_.

 Small interfering RNAs (siRNAs) targeting homo SMC1A and SNAIL were designed and purchased from General Biosystems (Anhui, China). siRNA and the negative control siRNA (siNC) were transfected at 20 nM in 6-well plates using Liopfectamine 3000 (Invitrogen, Carlsbad, USA) according to the manufacturer’s protocols. Total RNA and protein were extracted 48 h after transfection. The interference sequences were shown in Supplementary data Table 1.

SMC1A cDNA ORF clone (HG18194-UT) and SNAI2 cDNA ORF clone (HG11196-M) were purchased from SinoBiological(Beijing, China), and subcloned into a pCDNA3.1 vector. Plasmids were transfected into cells using Lipofectamine 3000 (Thermo Fisher, USA) following the manufacturer’s instructions. Cells were harvested 48 h after transfection.

### RNA isolation and reverse transcriptionquantitative PCR(RTqPCR)

Total RNA was isolated using TRIzol Reagent (TIANGEN, Beijing, China), and reverse transcription was performed using the Revertaid First Strand cDNA Synthesis Kit (Thermo Fish, Carlsbad, CA, USA) following the manufacturer’s instructions. The expression of mRNAs was quantified by RT-qPCR analysis with SYBR Green PCR Kit(Invitrogen, California, USA). β-actin was used as the normalization control, and expression levels were calculated based on the 2^−ΔΔCT^ method. The sequences of primers were shown in Additional file 1: Table 1.

### Western blot analysis

Total proteins were isolated from GC tissues and cells, and the concentration was measured with the BCA Kit (ThermoFisher, USA). Proteins were separated by 10% SDS-PAGE gel and transferred to 0.45 μm polyvinylidene difluoride (PVDF) membrane (Millipore, USA). After blocking with 5% non-fat dry milk for 2 h at room temperature, membrances were incubated with primary antibodies against SMC1A (1:2000, Immunoway), SNAIL (1:1000, CST), E-cadherin (1:2000, Proteintech), N-cadherin(1:1000, Proteintech), Vimentin (1:2000, Proteintech) overnight at 4 °C, followed by incubating with HRP-labeled secondary antibody. The bloting was visualized using an enhanced chemiluminescence reagent (Thermofisher, USA). β-actin was used as a loading control.

### Cell proliferation assay

Cell proliferation was assessed using the Cell Counting Kit-8 (CCK-8) assay (Beyotime, China). AGS or HGC27 cells were seeded separately into 96-well plates with the density of 4 × 10^3^ cells/well. On the second day, the medium was removed. Subsequently, 100 µl of basic RPIM 1640 medium or basic F12K medium with 10 µl CCK8 were added into HGC27 and AGS cells, respectively. Cells were then incubated for another 2 h, and the optical density was measured by a microplate Reader (Victor3 1420 Multilabel Counter, Perkin Elmer, USA) at 450 nm.

### Colony formation assay

AGS or HGC27 cells were seeded in 6-well plates at a density of approximately 1000 cells/well and allowed to grow for 14 days. Then the cells were washed triple with phosphate buffer, fixed in 4% paraformaldehyde (Solarbio, Beijing, China) 30 min, and then stained with 0.5% crystal violet (Solarbio, Beijing, China).

### Cell invasion assay

Cell invasion assays were performed in a transwell chamber covered with Matrigel (BD Biosicences, Bedford, USA). After 48 h of transfection, 1 × 10^5^ AGS or HGC27 cells were plated in the upper chambers with serum-free medium. By contraries, medium with 20% FBS were added into the lower chambers. After 48 h incubation, the invading cells were fixed in 4% paraformaldehyde and stained with 0.5% crystal viole.

### Wound healing assay

AGS and HGC27 cells were cultured in 6-well plates. When cells grew to about 80% confluence, cells were scratched by 10 µl plastic pipette tip. Next, cells were washed with PBS to remove debris. The wound-healing distance was measured at the 0 h and after 24 h to statistical cell migration.

### In vivo tumorigenesis assays

The animal assays were executed in compliance with the institutional ethics guidelines for animal experiments, which was approved by the committee on the Ethics of Animal Experiments of the Second Xiangya Hospital. A total of 10 BALB/C nude mice (4 weeks old, 18–22 g, five mice per group) were purchased from Hunan STA Laboratory Animal CO., Ltd (Changsha, China) and housed in specific pathogen-free (SPF) environment, with normal circadian rhythm of water and food intake. Animals were parandomly divieded into two groups: (1) vector group (injected with AGS cells transfected with control vector), (2) SMC1A group (injected with AGS cells transfected with SMC1A overexpression plasmids). About 0.2 ml cell suspension (1 × 10^7^cells /ml) were injected subcutaneously into the left axilla of nude mice. Tumor growth was calculated by measuring the length (a) and the width (b) diameter of the tumor with calipers every week, and the the tumor volume (V) was calculated using the formula V = (S^2^ × L)/2. Four weeks later, all the mice were euthanized and subcutaneous tumors were harvested.

### Immunofluorescence

Cells on sterile slips were fixed in 4% parafomaldehyde for 15 min and permeabilized with 0.1% Triton X-100 for 10 min at room temperature. Next, the slips were incubated with E-cadherin (1:50, #20874-1-AP, Proteintech) or Vimentin (1:100, #60330-1-Ig, Proteintech) antibody overnight at 4 °C. Subsequently, the slips were incubated with FITC labeled goat anti-rabbit IgG (1:1000, #ab6717, Abcam) or Cy3-AffiniPure Goat Anti-Mouse IgG (1:500, #115-165-003, Jackson) for 1 h. DAPI was used for nuclear staining. Finally, fluorescence was imaged under the fluorescent microscope (IX71, Olympus, Japan).

### Statistical analysis

Data are analyzed by the GraphPad Prism 7.0. The difference among groups was determined by T-test. All the experiments were repeated at least three times. *P* < 0.05 was considered as statistically significant.

## Results

### Overexpression of SMC1A in human gastric cancer

**Fig. 1:**
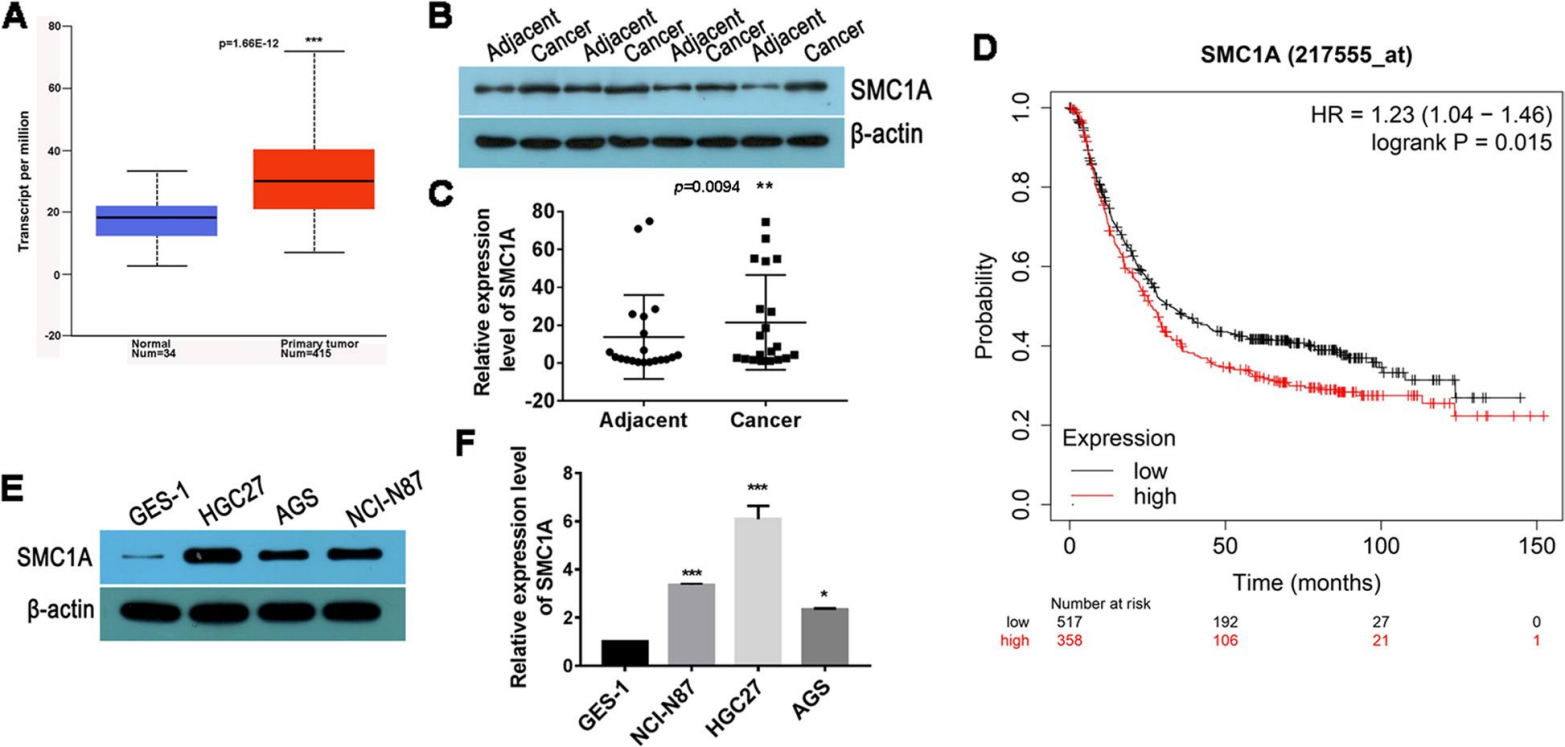
SMC1A was overexpressed in human gastric cancer. **A** The expression of SMC1A was analyzed in cancer tissues from TCGA data. blue: adjacent normal tissues and red: gastric cancer tissues. Western blot (**B**) and RT-qPCR (**C**) analysis for SMC1A mRNA in 20 samples of GC tissues and the corresponding adjacent tissues. **D** The overall survival of GC patients was evaluated using Kaplan-Meier Plotter. The red line represents SMC1A high expression group, and black line represents SMC1A low expression group. The expression of SMC1A was examined in human gastric cancer cell lines and the human gastric epithelial cell line GES-1using RT-qPCR method (**E**) and western blot method (**F**). **P < 0.01, **P < 0.001, ***P < 0.001

To investigate the characteristics in gastric cancer, we analysed SMC1A in the TCGA stomach adenocarcinoma database. As shown in Fig. 1A, SMC1A expression was evidently higner in tumor tissues compared with paracancerous tissues (Nomal). We further examined the expression of SMC1A in fresh tissue samples from 20 gastric cancer patients and found that SMC1A expression was higher in cancer tissues than that in corresponding adjacent tissues (Fig. 1B, C). Notably, high expression of SMC1A was associated with the poor overall survival in GC patients from Kaplan-Meier Plotter (http://kmplot.com/analysis/) (Fig. 1D). We also assessed the expression of SMC1A mRNA and protein in human gastric cancer cell lines (AGS, HGC27 and NCI-N87) and the human gastric epithelial cell line GES-1. As shown in Fig. 1E, F, SMC1A was highly expression in GC cell compared wih GES-1. These results suggesedt that SMC1A may play a role in the development and progression of gastric cancer.

### SMC1A promoted gastric cancer cell proliferation, invasion and migration

**Fig. 2:**
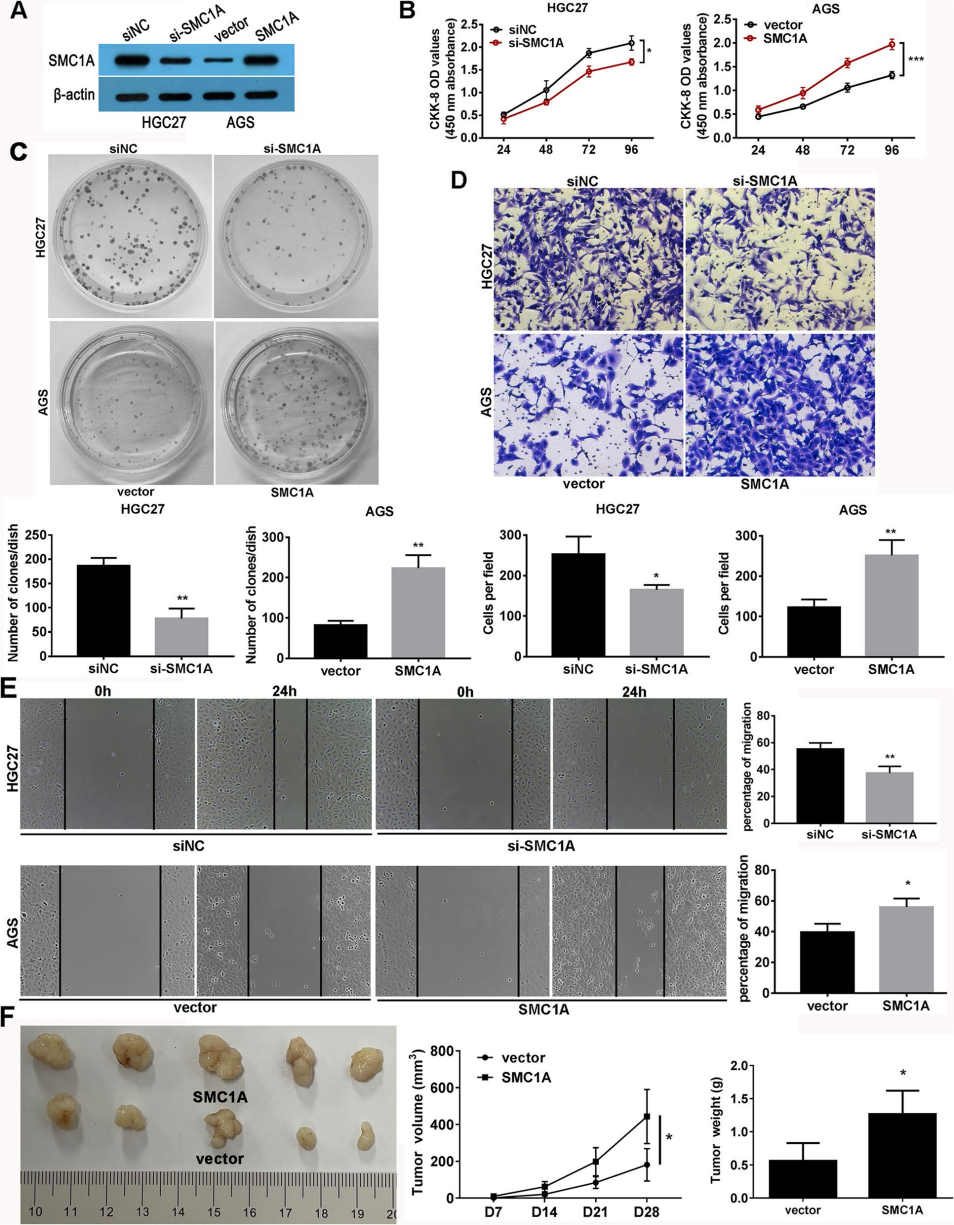
SMC1A promoted gastric cancer cell proliferation, invasion and migration. **A** The expression of SMC1A was examined in SMC1A silenced and overexpressed cells by western blot method. CCK-8 assay (**B**) and Colony formation assay (**C**) were used to determine the effect of SMC1A knockdown and overexpression on cell proliferation. Matrigel invasion assay (**D**) and Wound healing assay (**E**) analyzed the effect of SMC1A knockdown and overexpression on cell invasion and migration respectively. **F** Subcutaneous xenografts of AGS cells transfected with SMC1A overexpression plasmids. Images of tumors from nude mice at autopsy are presented (left), the tumor volumes were measured at the indicated time points (middle), and the average weight of the xenografted tumors was measured (right). *P < 0.05, **P < 0.01, ***P < 0.001

To investigate the functional role of SMC1A in GC, we used AGS and HGC27 cells as gain-of-function and loss-of-function models, respectively. We confirmed successful knockdown or overexpression of SMC1A using western blotting (Figs. 2A and S1A). CCK-8 assays displayed that depletion of SMC1A significantly reduced proliferation of HGC27 cells, while overexpression of SMC1A promoted proliferation of AGS cells (Figs. 2B and S1B). Colony formation assays also confirmed that SMC1A positively regulated colony formation ability in both HGC27 and AGS cells (Figs. 2C and S1C). Transwell invasion assays showed that SMC1A depletion decreased the invasion in HGC27 cells, while SMC1A overexpression increased invasion in AGS cells (Figs. 2D and S1D). Similarly, wound healing assays demonstrated that SMC1A overexpression promoted migration of AGS cells, while SMC1A knockdown inhibited migration of HGC27 cell (Figs. 2E and S1E). We further validated the effects of SMC1A overexpression on tumor growth in vivo by subcutaneously inoculating AGS cells transfected with either blank vector or SMC1A overexpression vector into nude micein AGS cells on the growth of human GC xenograft formation in nude mice. As shown in Fig. 2F, the tumour sizes and volume in SMC1A-overexpressing group were larger than those in the vector group. Meanwhile, a greater tumor weight was found in SMC1A-overexpressing group. These data indicated that SMC1A could significantly enhance human GC xenograft growth in nude mice. Taken together, these results suggest that SMC1A promotes proliferation, migration, and invasion of gastric cancer cells.

### SMC1A promoted EMT via upregulating SNAIL

**Fig. 3:**
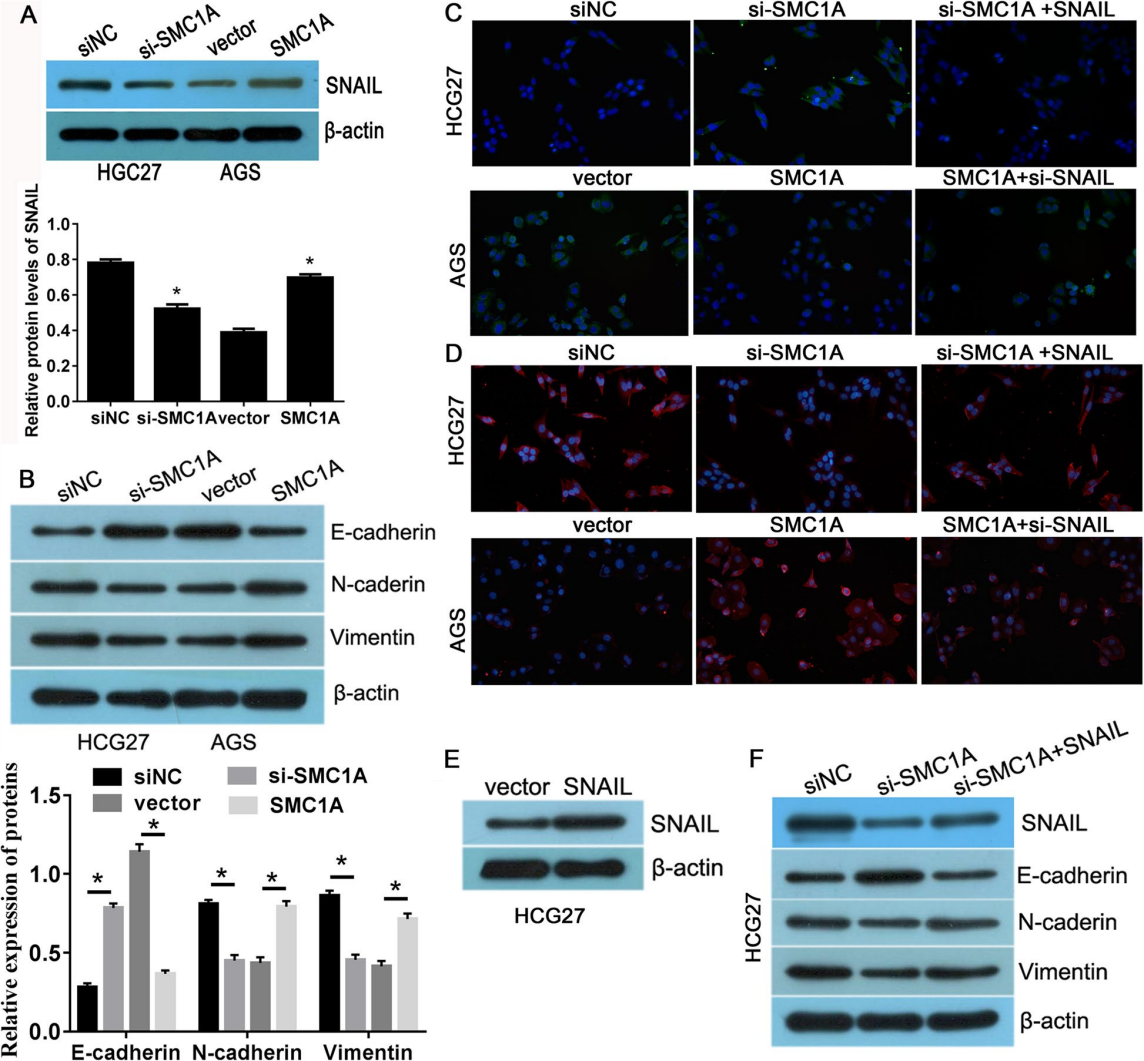
SMC1A promoted GC epithelial-mesenchymal transition (EMT) via upregulating SNAIL.** A** SNAIL level was examined in SMC1A silenced and overexpressed cells using western blot method. **B** Proteins level of EMT markers E-cadherin, N-cadherin and Vimentin were detected after SMC1A depletion and overexpression. Immunofluorescence assay detected the expression of E-cadherin (**C**) and Vimentin (**D**) in SMC1A silenced and overexpressed cells. **E** The expression of SNAIL was examined in SNAIL overexpressed cells by western blot method. **F** Proteins level of EMT markers E-cadherin, N-cadherin and Vimentin were detected in response to the treatment of SMC1A siRNA and SMC1A siRNA + SNAIL

Epithelial-mesenchymal transition (EMT) is a process which epithelial cells tansform inton a mesenchymal phenotype, concomitantly reduce the expression of E-cadherin regulated by one or several factors [17]. Snail family transcriptional repressor 1 (SNAIL) belongs to Snail family of zinc-finger transcription factor, which regulates EMT via directly suppressing epithelial marker E-cadherin or elevating mesenchymal markers [18–20]. Recently, Zhang et al. [21] have demonstrated that SMC1A regulated SNAIL expression via binding to the recognition site in the promoter of the SNAIL gene in breast cancer cells. Thus, we speculated that SMCIA may involves in EMT process via regulation SNAIL. As showing in Fig. 3A, SMC1A knockdown obviously decreased SNAIL expression, while SMC1A overexpression elevated SNAIL level. The detectation of EMT related marker showed that the expression of mesenchymal markers N-cadherin and Vimentin was reduced and epithelial marker E-cadherin was increased in SMC1A depletion cells. Converse results were observed in the SMC1A overexpression cells (Fig. 3B). Besides, we also analyzed the expressipn of EMT markers in tumor samples from nude mice. The result presented that SMC1A overexpression increased the expression of SNAIL, N-cadherin and Vimentin, but reduced the expression of E-cadherin (Fig. S2). Immunofluorescence (IF) assay further confirmed the reduced expression of vimentin and the increased expression of E-cadherin in SMC1A depletion cells, while an opposite result was presented in SMC1A-overexpressed cells (Fig. 3C and D). Furthermore, restoring SNAIL expression in SMC1A silenced cells or suppressing SNAIL expression in SMC1A overexpressed cells could significant mitigated the expression change of EMT related markers (Figs. 3E, F and S3A, B). This change for the expression of E-cadherin and Vimentin was also verified by IF assay (Fig. 3C and D). These results implied that SMC1A facilitated EMT via upregulating SNAIL.

### SMC1A promoted GC cell proliferation, invasion and migration via SNAIL

**Fig. 4:**
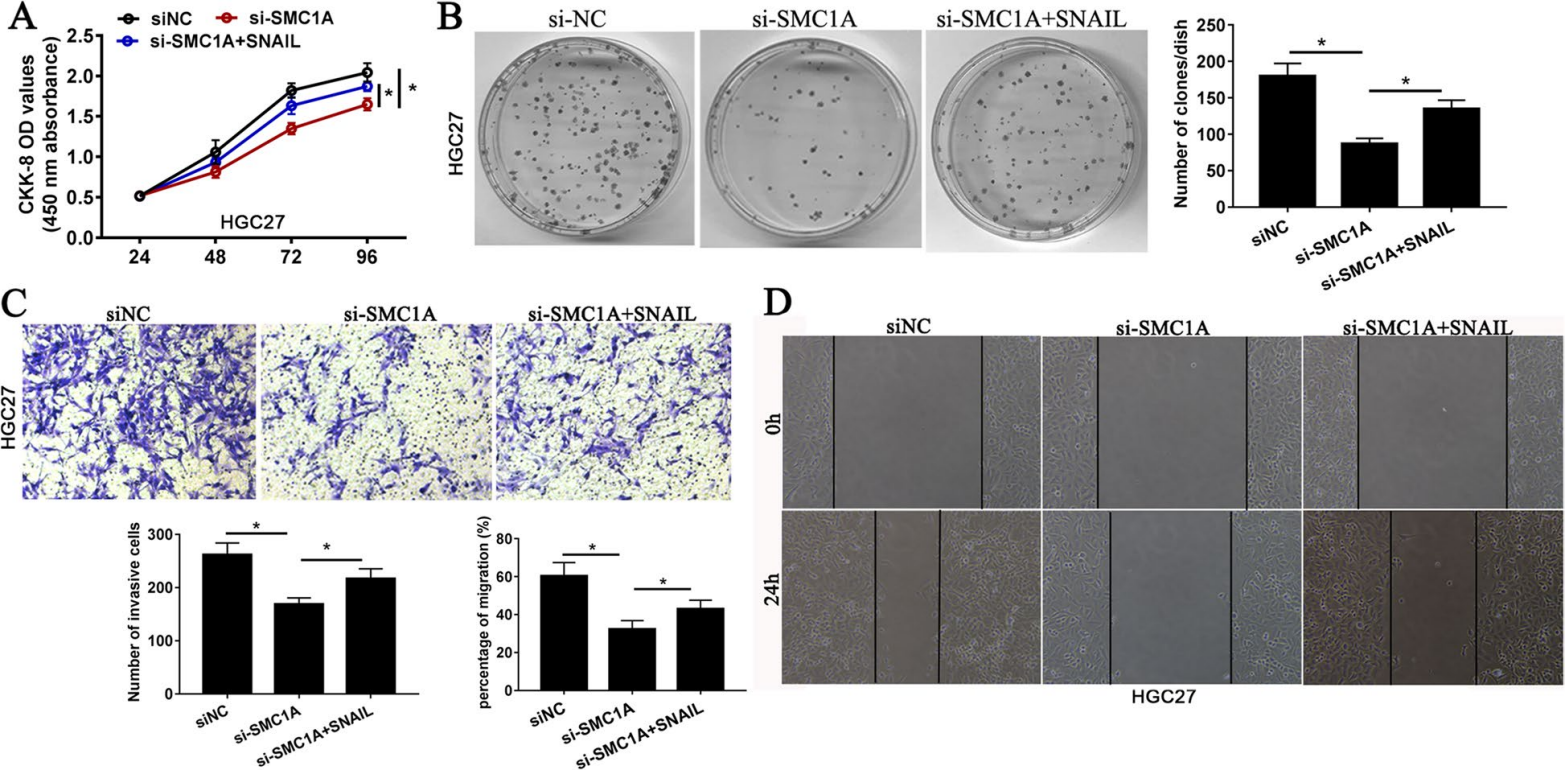
SMC1A promoted GC cell proliferation, invasion and migration via SNAIL. CCK-8 assay (**A**) and Colony formation assay (**B**) were performed to analysis cell proliferation in response to the treatment of SMC1A siRNA and SMC1A siRNA + SNAIL. Matrigel invasion assay (**C**) and Wound healing assay (**D**) used to investigate cell invasion and migration in response to the treatment of SMC1A siRNA and SMC1A siRNA + SNAIL. *P < 0.05

Numerous literature have been reported that EMT may promotes cell growth, migration and metastatic in GC [22–24]. Therefore, rescue experiments were performed to evaluate whether SMC1A promoted GC biology behaviors via EMT regulator SNAIL. As determined by CCK-8, clone formation, Transwell invasion and wound-healing assays, restoring SNAI2 expression could evidently attenuated the suppressive effect on GC cell proliferation, clone formation, invasion and migration causing by SMC1A silencing (Fig. 4A-D). Meanwhile, suppressing SNAI2 expression could evidently attenuated the promotive effect on GC cell proliferation, clone formation, invasion and migration causing by SMC1A overexpression (Fig. S3C-F). It’s indicated that SMC1A promoted GC cell proliferation, migration and invasion via SNAIL.

## Discussion

Gastric cancer is a significant health concern worldwide, affecting nearly 2 million individuals annually. Unfortunately, 75–85% of those diagnosed with gastric cancer will die within 5 years, making it the third most fatal cancer globally [25]. Currently, inadequate early detection and the tumor’s tendency to invade and metastasize pose significant challenges to effective treatment for gastric cancer. Although treatments such as chemotherapy, radiotherapy, palliative resection, and gastrectomy have improved outcomes, the prognosis remains poor [26]. Therefore, there is an urgent need to explore the molecular mechanisms underlying the initiation and progression of gastric cancer to improve treatment strategies and monitor prognosis. In this study, we found that SMC1A was upregulated in GC tumor tissues and cells, and high expression of SMC1A was associated with the poor overall survival. Our cell function and mechanism assays revealed that SMC1A facilitates gastric cancer cells proliferation, migration and invasion via promoting SNAIL activated EMT.

SMC1A functions in a variety of biological activities as a critical structural maintenance component of chromosomal proteins [11]. SMC1A is critical for the development and spread of tumors, according to prior studies [8]. SMC1A has been identified as a proto-oncogene in the majority of cancers that may facilitate the growth and spread of cancer [10, 12]. As reported in CRC, the high expression of SMC1A promotes liver metastasis by recruiting the tumor-associated-fibroblastas (TAFs) to facilitate prophase tumor construction and tumorigenicity [14]. However, SMC1A is downregulated in acute myeloid leukemia, and its low expression indicates a poor prognosis. Overexpression of SMC1A has been linked to apoptosis [15, 27]. Our research discovered that SMC1A might promote GC cell proliferation, migration and invasion in vitro, which is consistent with the majority of literature. The most common reason for mortality in cancer patients is metastasis, a multi-step, complicated. To spread from the original tumors and enter into the circulation during carcinoma metastasis, stationary epithelium-derived tumor cells must first become migratory and invasive [28]. As a result, tumor metastasis requires the movement of malignant cells.

EMT was originally identified during embryonic development and was critical for the development of germ layers and organs [29]. Epithelial cells lose their polarity and intrecellular adhesion during EMT process, and acquire various of mesenchymal phenotypes, including motility and invasiveness [30]. The group of EMT transcription factors (EMT-IFs), which includes Zeb, SNAIL, Slug and Twist, as well as the epithelial marker E-cadherin and the mesenchymal markers N-cadherin and Vimentin, are responsible for inducing this process [31]. Accumulated evidence has revealed that EMT is involved tumor invasion and metastasis [29, 32, 33]. As the essential epithelial marker of EMT that is located in epithelial cell junctions and takes part in the construction of intercellular adhesion complexes, the decrease of E-caherin expression is one of the key characteristics of EMT [29]. When E-cadherin is eliminated, the bonds between cells become less rigid. Simutaneously, cells adhesion is reduced, which makes cells more likely to detach from the primary tumor site and enhances their mobility and invasive capacity [29, 34]. It’ well established that several critical signaling pathways, including transforming growth factor beta (TGFβ), Wnt, Notch and Hedgehog are involved in EMT [35]. In the TGFβ signal pathway, activated SMAD2 and SMAD3 form a complex with SMAD4, which then translocates to the nucleus and increases the transcription of EMT-TFs [36]. In the Wnt signalling, it suppresses the degradation complex component glycogen synthase kinase 3 beta (GSK-3β) through Disheveled (DSH), leading to the translocation of β-catenin into the nucleus and enhances the transcription of Snail [37]. Upon activation of the Notch pathway, the Notch intracellular fragment is released through a cascade of proteolytic cleavages and activates CSL family of transcription factors to upregulate EMT-TFs [38]. Through activated GLI family transcription factors upregulating snail expression, Sonic Hedgehog signaling may cause EMT [39]. These signaling pathways control several EMT-TFs to mediate EMT activities. In a prior work, it was shown that SMC1A controlled EMT to mediate prostate cancer radioresistance [10]. In our study, we also found that SMC1A might cause EMT in GC. It is plausible that SMC1A has a role in the chemoresistance of GC cells given the growing body of research demonstrating that cancer cells undergo EMT and contribute to this resistance [40–42], However, investigations are required to validate this idea. The mechanism of SMC1A modulation in EMT is currently unknown. SNIAIL, also named SNAI1, is an essenial EMT inducible factor that suppressses E-cadherin transcription by interacting with the conserved E-box motif in the promoter region [43]. Notably, it has been reported that SMC1A may play a role in EMT by controlling SNAIL. This is because SMC1A has been shown to bind to the promoter region of the SNAIL gene in breast cancer cells [21]. In this study, we discovered that SMC1A might stimulate EMT and malignant cell behaviors via regulating SNAIL, which sheds light on the oncogenic function of SMC1A in GC.

## Conclusion

In summary, our study revealed SMC1A facilitates gastric cancer cell proliferation, migration and invasion via promoting SNAIL activated EMT, which indicated SMC1A may be a potential target for gastric cancer therapy.

### Supplementary Information

**Additional file 1.**

**Additional file 2.**

## Data Availability

The data used to support the findings of this study are available from the corresponding author upon request.

## Abbreviations

SMC1A: Structural maintenance of chromosomes protein 1A
GC: Gastric cancer
EMT: Epithelial-mesenchymal transition
SNAIL (SNAI1): snail family transcriptional repressor 1
GEO: Gene expression omnibus
EGA: The European Genome-phenome Archive
TCGA: The Cancer Genome Atlas
FBS: Fetal bovine serum
ORF: Open reading frame
RTqPCR: Reverse transcriptionquantitative PCR
PVDF: Polyvinylidene difluoride
CCK-8: Cell Counting Kit-8
TAFs: Tumor-associated-fibroblastas
EMT-IFs: EMT transcription factors
Zeb Zinc: finger E-box binding homeobox
TGFβ: Transforming growth factor beta
SMAD2,3,4: SMAD family member 2,3,4
GSK-3β: glycogen synthase kinase 3 beta
DSH: Disheveled
SHH: Sonic Hedgehog
SMO: Smoothened, frizzled class receptor
