# Supplementary figures and images for: SMC1A facilitates gastric cancer cell proliferation, migration, and invasion via promoting SNAIL activated EMT
Source: BMC Gastroenterol. 2023 Aug 4;23:268. doi: 10.1186/s12876-023-02850-z (PMC10401881; doi:10.1186/s12876-023-02850-z)

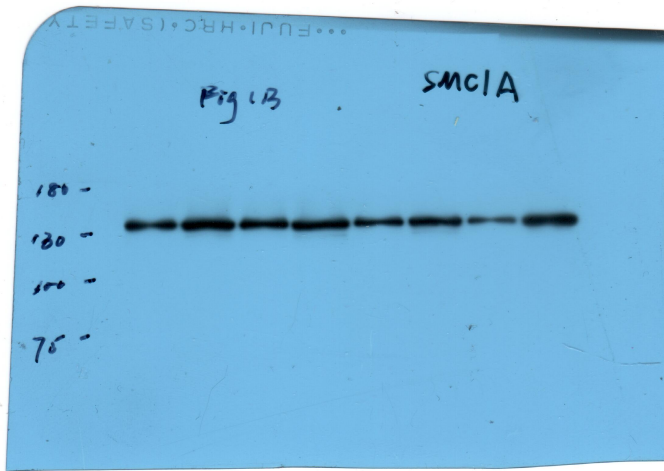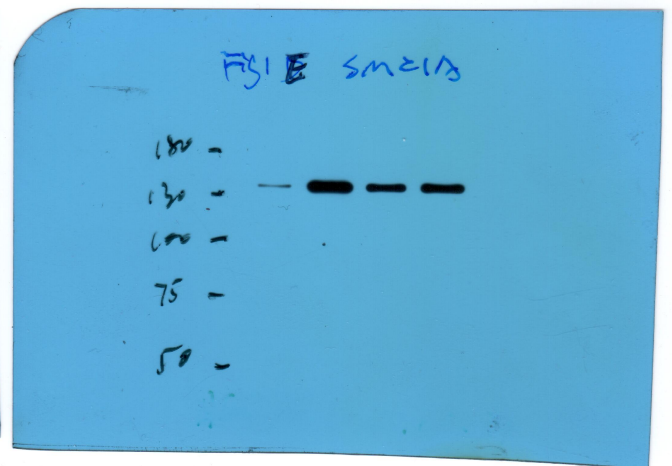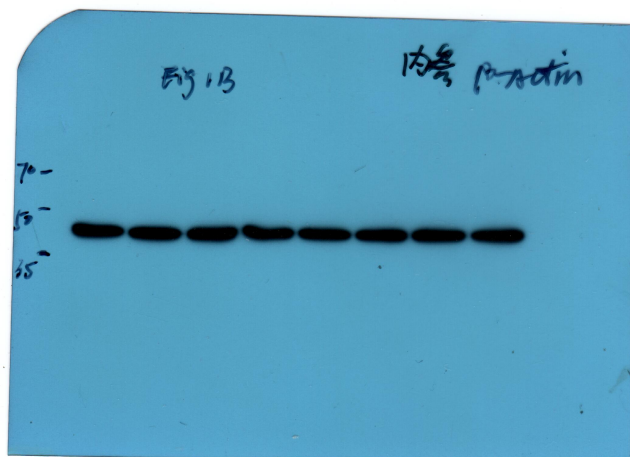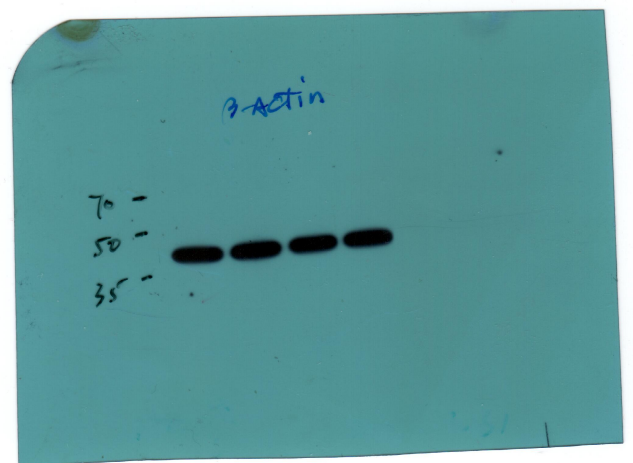

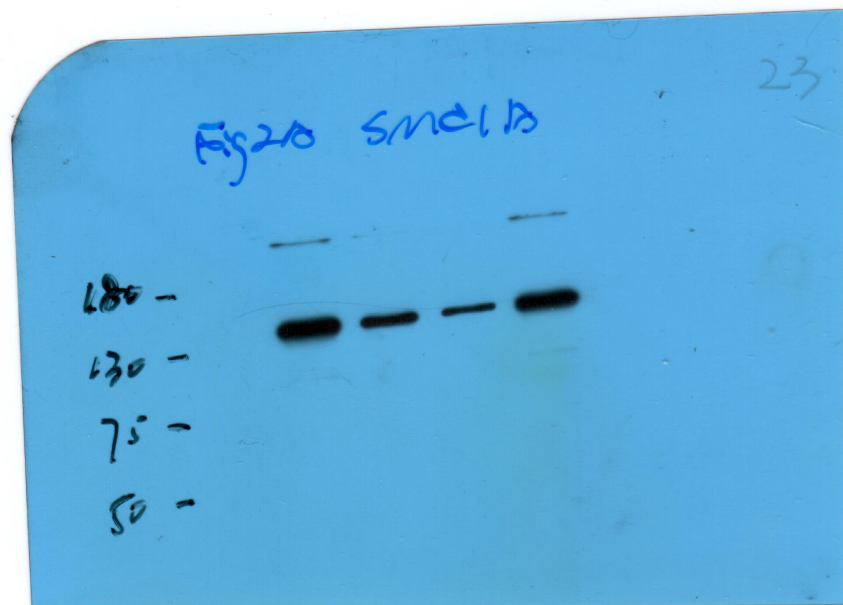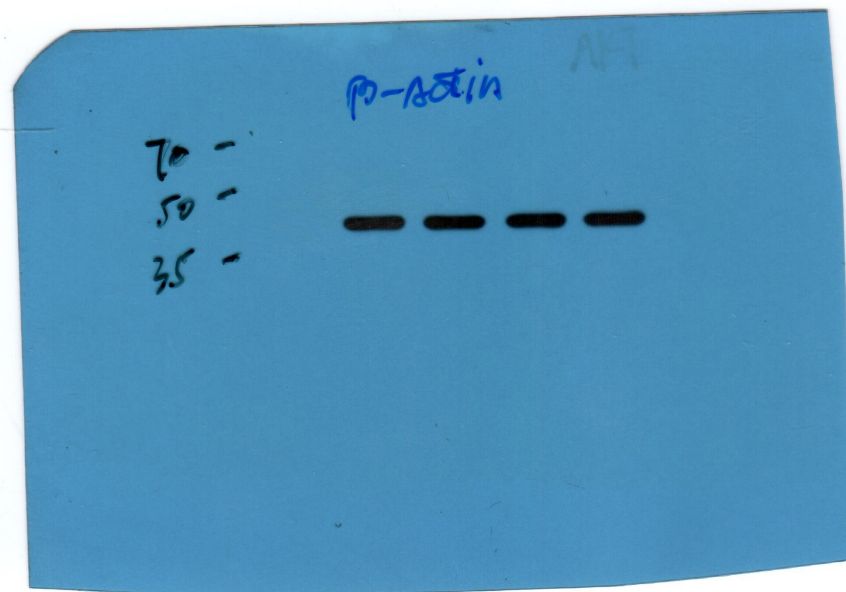

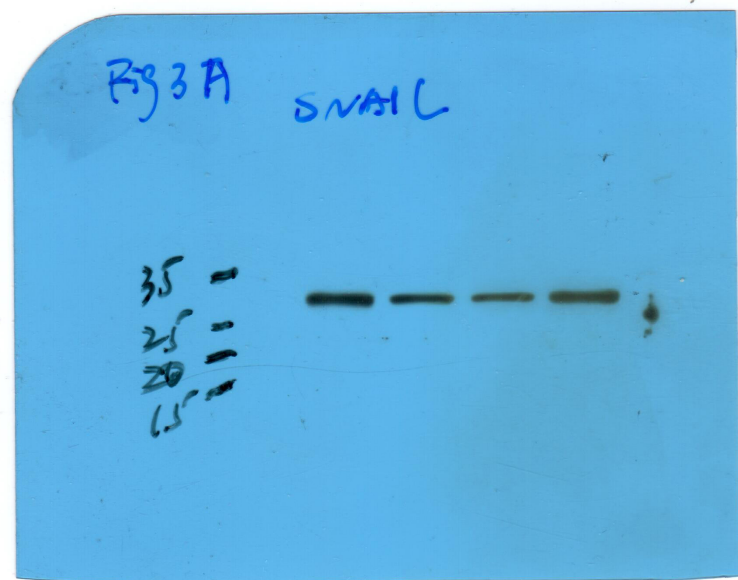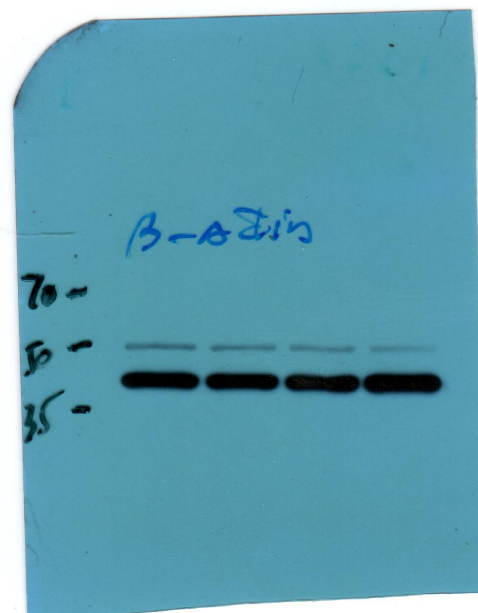

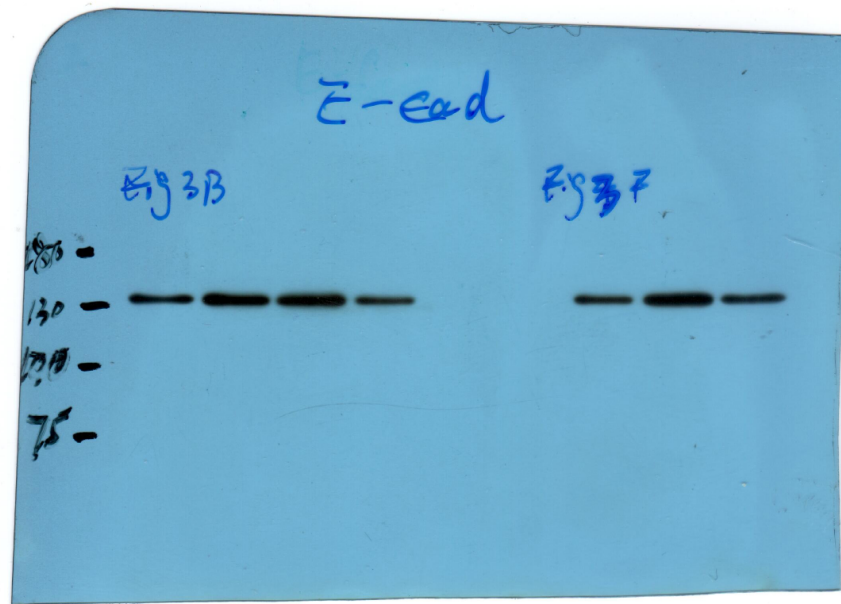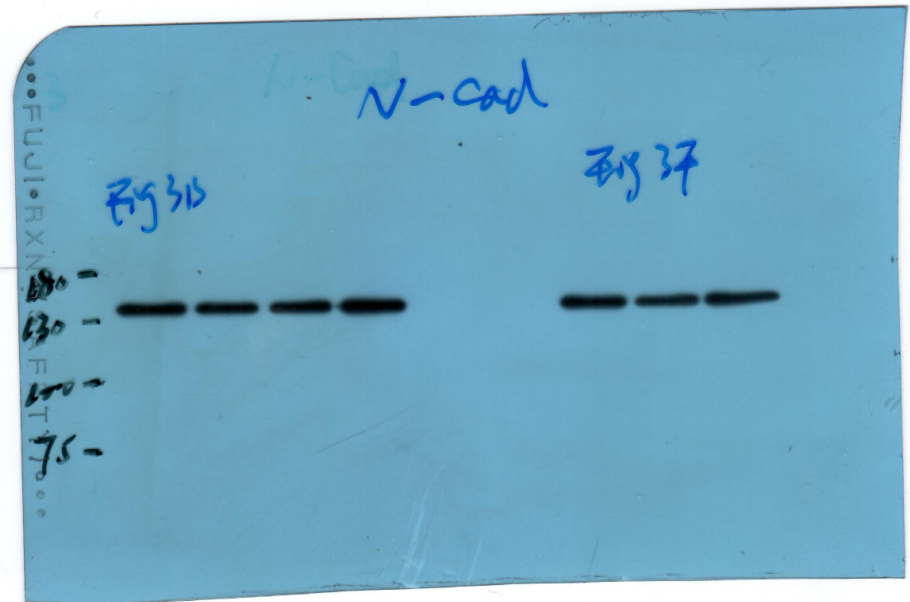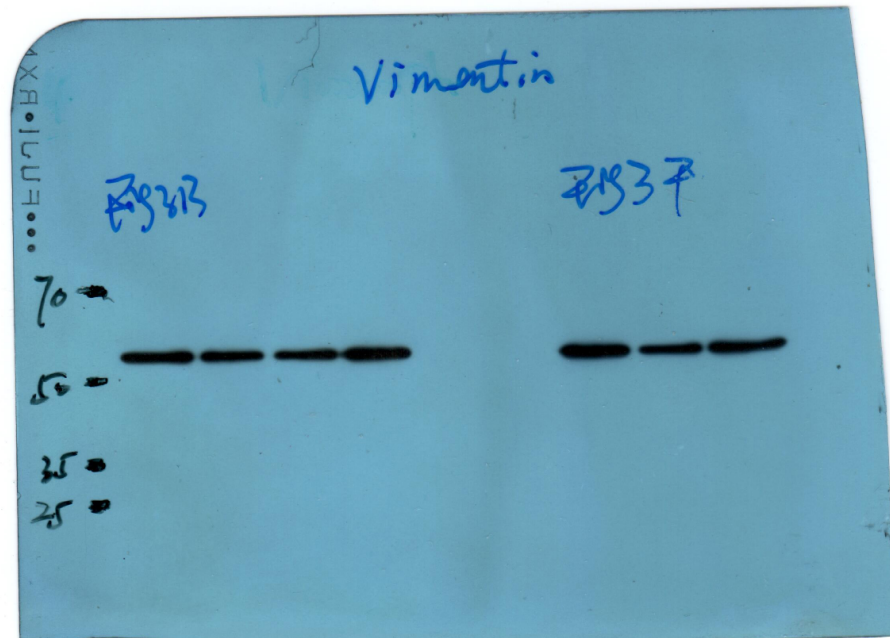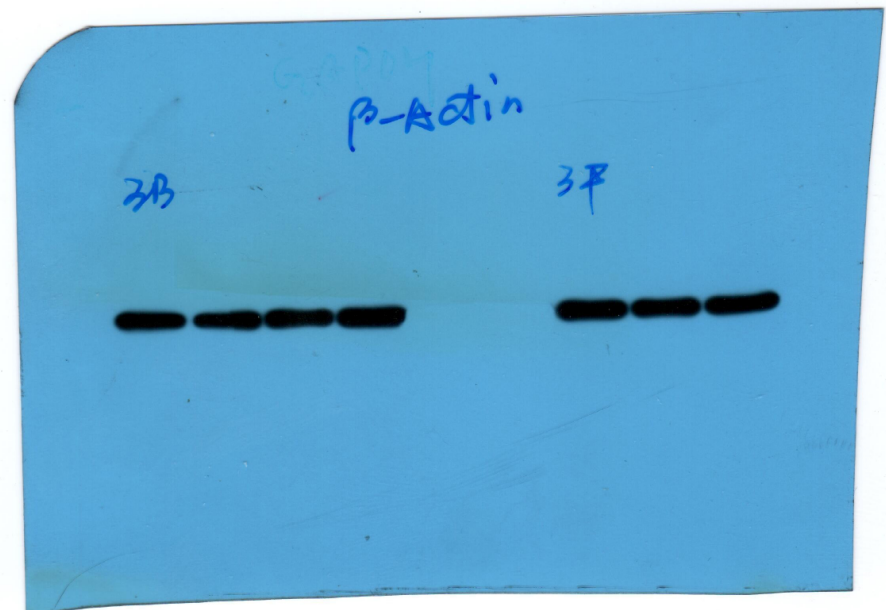

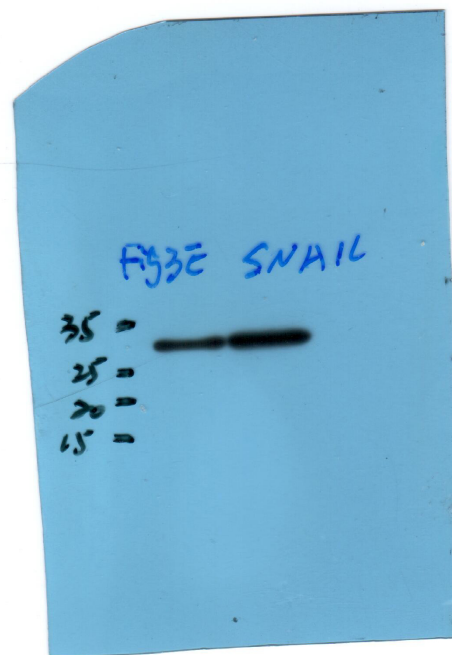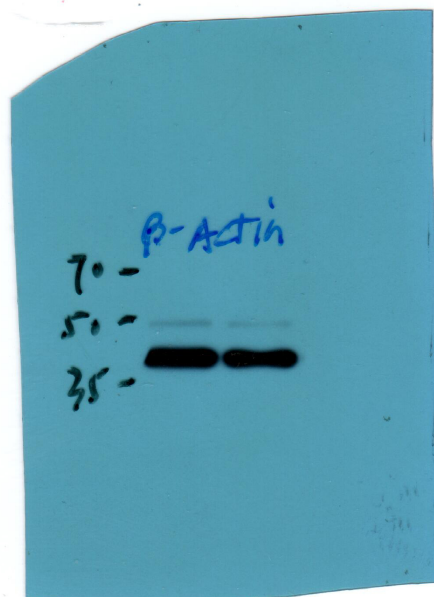

Supplement: Supplementary file 1 — Additional file 1. [file 12876_2023_2850_MOESM1_ESM.pdf]
