# Supplementary material for: SMC1A facilitates gastric cancer cell proliferation, migration, and invasion via promoting SNAIL activated EMT
Source: BMC Gastroenterol. 2023 Aug 4;23:268. doi: 10.1186/s12876-023-02850-z (PMC10401881; doi:10.1186/s12876-023-02850-z)
Supplement: Supplementary file 2 — Additional file 2. [file 12876_2023_2850_MOESM2_ESM.docx]

supplementary data

Table1: The sequence information of siRNAs and primers

| ` | Sequence (5’ **‑** 3’) |
| --- | --- |
| SMC1A siRNA | sense: GCGGGAAAUUGAAGAGAAUTT |
|  | antisense: AUUCUCUUCAAUUUCCCGCTT |
| SMC1A siRNA-2 | sense: GGAGGUUCUUCUGAGUACATT |
|  | antisense: UGUACUCAGAAGAACCUCCTT |
| SNAIL siRNA | sense: AAUCGGAAGCCUAACUACATT |
|  | antisense: UGUAGUUAGGCUUCCGAUUTT |
| siNC | sense: UUCUCCGAACGUGUCACGUTT |
|  | antisense: ACGUGACACGUUCGGAGAATT |
| SMC1A | sense: CATCAAAGCTCGTAACTTCCTCG |
|  | antisense: CCCCAGAACGACTAATCTCTTCA |
| β-actin | sense: TTCCTTCCTGGGCATGGAGTC |
|  | antisense: TCTTCATTGTGCTGGGTGCC |

supplementary figures


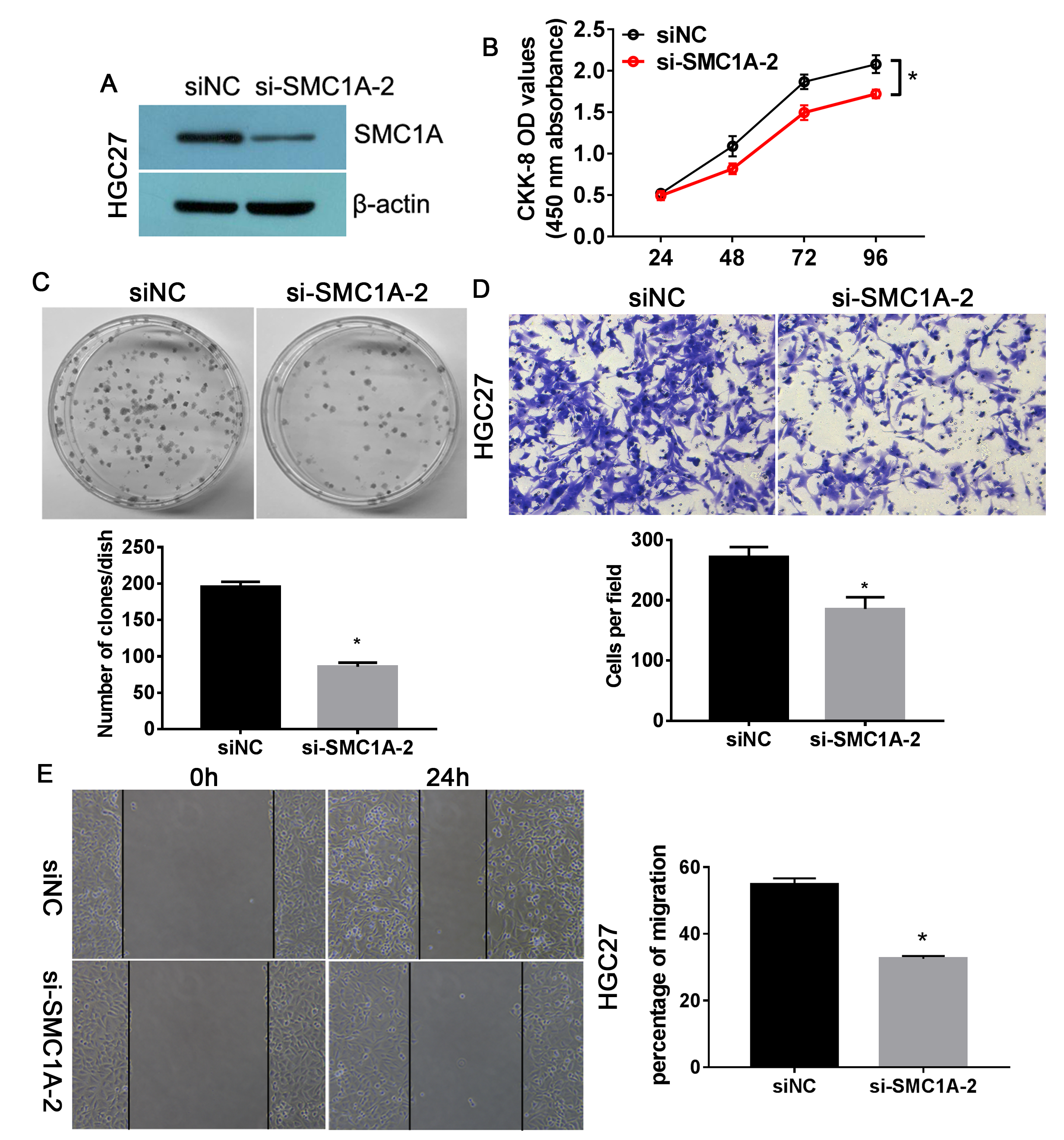


**Figure S1. SMC1A depletion inhibited gastric cancer cell proliferation, invasion and migration.** (A) Western blot detected the interference efficiency of si-SMC1A-2 in HGC27 cells. CCK-8 assay (B) and Colony formation assay (C) were used to determine the effect of SMC1A knockdown on HGC27 cell proliferation. Matrigel invasion assay (D) and Wound healing assay (E) analyzed the effect of SMC1A knockdown on HGC27 cell invasion and migration respectively. *P<0.05.


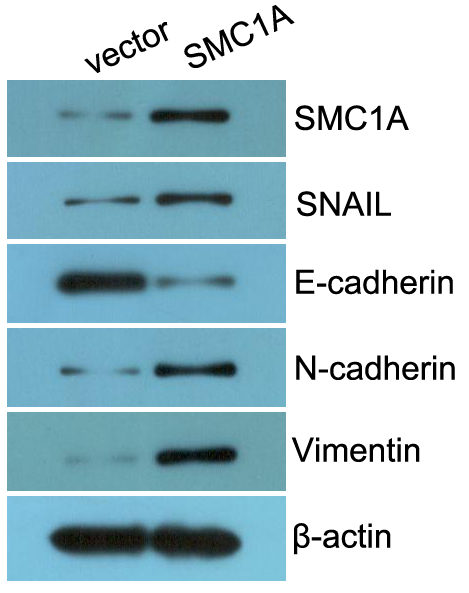


**Figure S2. Western blot detected the expression of SNAIL as well as EMT markers in tumor samples from nude mice.**


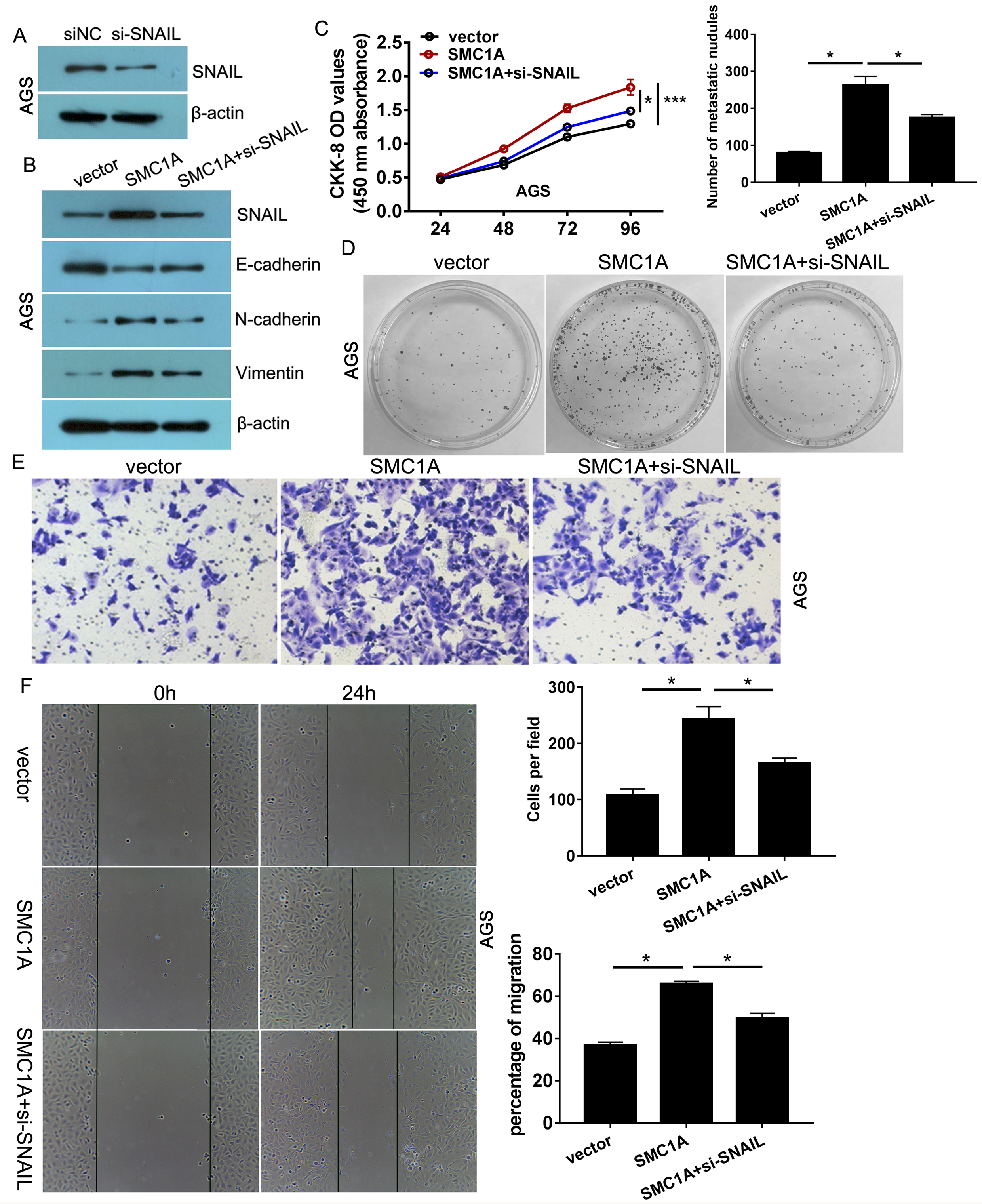


**Figure S3. SMC1A promoted GC cell proliferation, invasion and migration via SNAIL.** (A) The expression of SNAI2 was examined in SNAIL silenced AGS cells by western blot. (B) Proteins level of EMT markers E-cadherin, N-cadherin and Vimentin were detected in response to the treatment of SMC1A and SMC1A+ si-SNAIL. CCK-8 assay (C) and Colony formation assay (D) were performed to analysis cell proliferation in response to the treatment of SMC1A and SMC1A+ si-SNAIL. Matrigel invasion assay (E) and Wound healing assay (F) used to investigate cell invasion and migration in response to the treatment of SMC1A and SMC1A+ si-SNAIL. *P<0.05.
